# The Successful Treatment of COVID-19-Induced Bullous Pemphigoid With Dupilumab

**DOI:** 10.7759/cureus.30541

**Published:** 2022-10-21

**Authors:** Michelle A Savoldy, Teja Tadicherla, Zade Moureiden, Noura Ayoubi, Brooke T Baldwin

**Affiliations:** 1 Dermatology, University of South Florida Morsani College of Medicine, Tampa, USA; 2 Dermatology, University of South Florida, Tampa, USA; 3 Dermatology, James A. Haley Veterans’ Hospital, Tampa, USA

**Keywords:** covid-19, off-label drug use, autoimmune blistering diseases, dupilumab, bullous pemphigoid

## Abstract

Bullous pemphigoid (BP) is a rare autoimmune blistering condition that predominantly affects the elderly population. Typical treatment regimens target the immune system and inflammatory response. We present a case of BP in a 78-year-old male patient that occurred following the coronavirus disease 2019 (COVID-19) vaccination. This case was refractory to topical steroids and immunosuppressants. However, it responded to treatment with dupilumab, a monoclonal antibody therapy. Dupilumab is classically indicated for the treatment of asthma, eosinophilic esophagitis, atopic dermatitis, and chronic rhinosinusitis with nasal polyposis. We highlight the importance of considering the off-label use of dupilumab and its success in treating BP.

## Introduction

Bullous pemphigoid (BP) is a rare autoimmune blistering disorder that primarily impacts patients 60 years and older. It can be triggered by various interventions that impact the immune system, such as medications, vaccines, infections, or trauma that disrupts the skin [1]. BP can initially manifest as an intensely pruritic eczematous-type rash and progress to large fluid-filled blisters [2]. Typical treatment regimens include immunosuppressants and anti-inflammatory medications [3]. We present a case of BP that appeared following the coronavirus disease 2019 (COVID-19) vaccination. The case was refractory to topical steroids and immunosuppressants; however, it responded to treatment with dupilumab, a monoclonal antibody therapy that functions to depress the body’s inflammatory response by inhibiting IL-4 and IL-13 through blockage of the IL-4 receptor alpha [4].

## Case presentation

**Figure 1:**
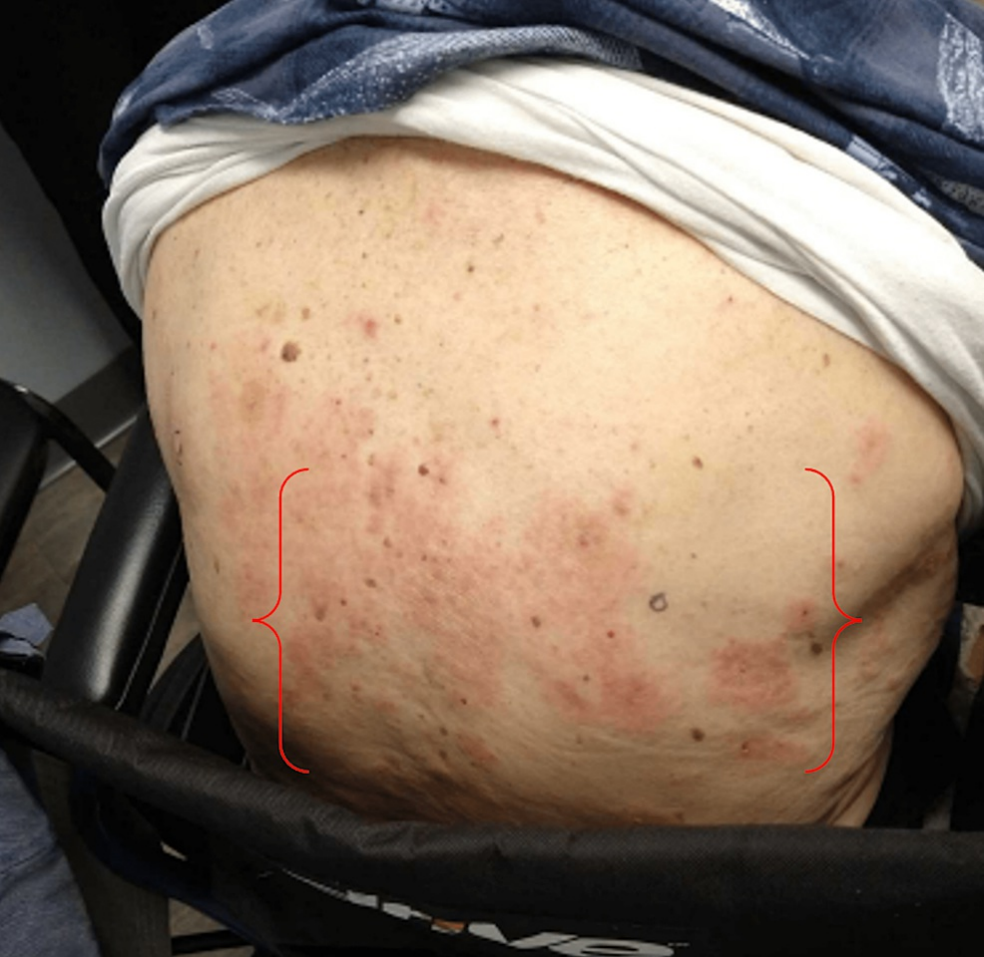
Initial presentation of BP as tender, pruritic, erythematous plaques on the back (red brackets) BP: bullous pemphigoid

**Figure 2:**
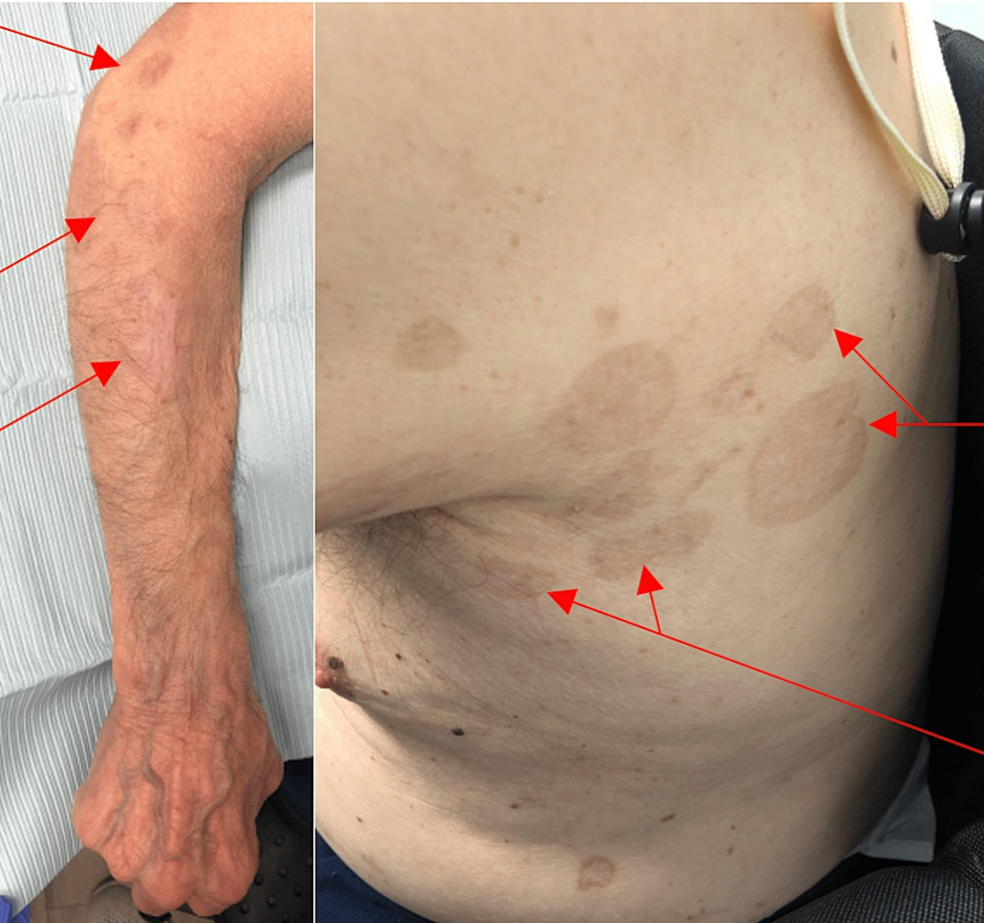
Progression to larger bullous lesions spreading from the back to the trunk and arms (red arrows)

A 78-year-old male with a past medical history significant for type 2 diabetes mellitus and neurocognitive disease presented with an extremely itchy and painful back rash one week after receiving the first dose of the COVID-19 vaccination in October 2021 (Figure 1). It was initially thought to be shingles. After receiving the second vaccination six weeks later, the rash progressed, with the forming of large, 5-10 cm blisters that spread from the back to the trunk, arms, and chest, with those in the underarms being the most severe. The blisters were filled with serosanguinous fluid and eventually resolved with hyperpigmentation (Figure 2).

**Figure 3:**
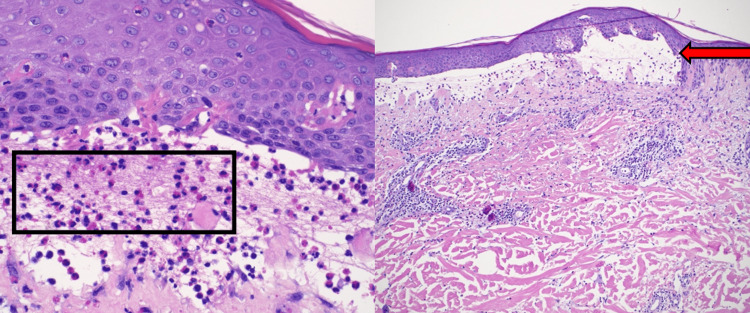
Pathology from biopsy-confirmed BP showing subepidermal blister (red arrow) with lymphocytic and eosinophilic infiltrate in the subepidermal cleft (black box) BP: bullous pemphigoid

At this point, it was thought to be contact dermatitis. The initial rash was treated with betamethasone dipropionate and triamcinolone 0.1% ointments, which soothed the pruritus but did not suppress blister formation. Topical triamcinolone and lidocaine only provided relief for the smaller blisters while the large ones were unaffected. Once the rash worsened, the patient was trialed with a two-week course of an oral prednisone taper and oral doxycycline 100 mg twice a day for two months. During this time, the patient was hospitalized with methicillin-resistant Staphylococcus aureus (MRSA) infection within the bullae, which was treated with intravenous vancomycin, discontinuation of doxycycline, and continuation of prednisone. The patient was diagnosed with biopsy-proven BP, which showed linear deposition of IgG and C3 along the basement membrane and eosinophils within the subepidermal cleft (Figure 3). Following the resolution of the MRSA infection, the patient agreed to trial dupilumab. Of note, the patient was taking several medications to treat his other chronic conditions throughout this time, including Levemir, Humalog, glimepiride, hydralazine, clonidine, terazosin, carvedilol, ezetimibe, atorvastatin, allopurinol, hydrocodone, and vitamin D. Dupilumab was initiated on December 22, 2021. He received one injection every two weeks. At the six-week follow-up, his condition had significantly improved, with only smaller, 1-2-cm-sized blisters appearing less frequently. These were responsive to topical betamethasone cream. While on dupilumab, he had only developed one larger blood-filled blister that had appeared during the treatment and had proceeded to self-resolve. He has not experienced any negative side effects while on dupilumab.

## Discussion

BP is an autoimmune condition of the skin characterized by subepithelial blisters. The pathology of BP involves an autoimmune antibody-mediated attack of the epithelial basement membrane. Specific targets include hemidesmosomal proteins BP 180 and BP 230. The predominant subclass of the antibody is IgG4 [5]. There is evidence that supports the role of Th2 cytokines, including IL-4 and IL-13, in the recruitment and adhesion of eosinophils within dermal infiltrates in blisters [6]. Clinically, this process can manifest with a prodromal phase that precedes bullae formation and may present as pruritic, eczematous-like lesions, followed by the formation of tense, fluid-filled blisters on the skin [2].

BP most commonly affects elderly patients above 60 years of age. It can arise in the setting of infection, medications, vaccines, and physical interventions such as those related to trauma, surgery, burns, and phototherapy [1]. As this is an autoimmune condition, numerous medical interventions that alter the balance of the immune system can contribute to the development of BP. For example, cancer patients receiving immunotherapy can develop bullous lesions after initiating treatment [7,8]. In the case presented here, the newly-developed COVID-19 vaccination triggered a BP eruption. Comorbidities include neurologic diseases, the strongest association being stroke, malignancies, other autoimmune conditions, and other dermatologic conditions, namely psoriasis [1].

Initial treatment of BP is typically oral or high-potency topical corticosteroids or doxycycline. Treatment selection depends on the severity of the condition and must be balanced with the side effect profile. When these typical treatment regimens do not work, biological therapies can be attempted, and while BP is not considered an official indication for these methods, there is increasing evidence from various case reports supporting their efficacy [9,10].

Dupilumab is a monoclonal antibody that inhibits the IL-4 receptor alpha, antagonizing IL-4 and IL-13 and in turn impeding the body’s inflammatory response. Its indications include asthma, eosinophilic esophagitis, atopic dermatitis, and chronic rhinosinusitis with nasal polyposis [4].

Dupilumab’s action against IL-4 and IL-13 makes it a possible therapeutic candidate for BP that is refractory to steroid therapy or doxycycline. Although it is not considered a first-line treatment for BP, it is important for providers to acknowledge the benefits that it can provide in allowing access to another pathway for immunosuppression and in turn leading to clinical improvement among these patients.

## Conclusions

BP is a rare autoimmune blistering disorder that predominately presents in adults older than 60 years of age. Because of its autoimmune nature, treatments are focused on hindering the immune response and decreasing inflammatory mediators. Typical treatments include high-potency corticosteroids or doxycycline. Long-term steroid exposure can place patients at increased risk for complications such as skin atrophy and infections. Treatment regimens must be selected to optimize the balance between potential risks and benefits. If typical regimens fail to treat BP, high-dose steroids should not be continued without benefit. The next step may include a trial of biological therapy. Dupilumab is a monoclonal antibody that inhibits inflammatory mediators IL-4 and IL-13. As there is potential for IL-4 and IL-13 to aid in eosinophilic deposition within bullous blisters, dupilumab can work effectively against BP, as shown in this case report. Beyond highlighting the efficacy of dupilumab in treating BP, this case report expands the spectrum of potential indications to also include autoimmune COVID-19-induced BP. Healthcare providers should be aware of the off-label use of this drug in the successful treatment of BP of various etiologies.
